# Immunotherapeutic Strategies for Prostate Cancer: A Comprehensive Review

**DOI:** 10.3390/cancers18020255

**Published:** 2026-01-14

**Authors:** Ana K. Flores-Islas, Cecilia Rico-Fuentes, Erick Sierra-Díaz, Mariel García-Chagollán, Ana Laura Pereira-Suárez, José Sergio Zepeda-Nuño, José M. Moreno-Ortiz, Adrián Ramírez-de-Arellano

**Affiliations:** 1 Instituto de Investigación en Cáncer e Infecciones, Departamento de Microbiología y Patología, Centro Universitario de Ciencias de la Salud, Universidad de Guadalajara, Guadalajara 44340, Jalisco, Mexico; ana.flores5605@alumnos.udg.mx (A.K.F.-I.); cecilia.rico9313@alumnos.udg.mx (C.R.-F.); ana.pereira@academicos.udg.mx (A.L.P.-S.); 2 Unidad Médica de Alta Especialidad, Hospital de Especialidades, Centro Médico Nacional de Occidente, Divisón de Epidemiología, Guadalajara 44340, Jalisco, Mexico; erick.sierra1353@academicos.udg.mx; 3 Departamento de Urología, Centro Universitario de Ciencias de la Salud, Universidad de Guadalajara, Guadalajara 44340, Jalisco, Mexico; 4 Instituto de Investigación en Ciencias Biomédicas, Centro Universitario de Ciencias de la Salud, Universidad de Guadalajara, Guadalajara 44340, Jalisco, Mexico; chagollan@academicos.udg.mx; 5 Centro de Investigación y Diagnóstico de Patología, Centro Universitario de Ciencias de la Salud, Universidad de Guadalajara, Guadalajara 44340, Jalisco, Mexico; jsergio.zepeda@academicos.udg.mx; 6 Instituto de Genética Humana “Dr. Enrique Corona Rivera”, Departamento de Biología Molecular y Genomica, Centro Universitario de Ciencias de la Salud, Universidad de Guadalajara, Guadalajara 44340, Jalisco, Mexico

**Keywords:** prostate cancer, immunotherapy, immune checkpoint inhibitors, adoptive cell therapy, CAR T-cell therapy, personalized medicine

## Abstract

Prostate cancer (PCa) is the leading cause of cancer-related deaths worldwide and the second most common cancer among men. Treatment options depend on factors like age, androgen sensitivity, PSA levels, Gleason score, TNM stage, and recurrence risk. Available treatments include hormonal therapy, radiation, surgery, and chemotherapy. Early immunological treatments were limited by poor lymphocyte infiltration and an immunosuppressive environment. Today, strategies such as dendritic cell vaccines, immune checkpoint inhibitors (ICIs), and adoptive cell therapy (ACT) are used. ACT, especially CAR T-cell strategies, aims to overcome traditional treatment limitations, particularly in advanced and metastatic castration-resistant prostate cancer (mCRPC), though it remains in early development. Personalized medicine uses molecular insights from the diseased tissue to tailor treatments. Variability in patient response, due to tumor heterogeneity and prior treatments, highlights the importance of personalized and combination therapies as future strategies for effective immunotherapy. This review explores the current landscape of PCa. We analyze treatment guidelines established by NCCN and EANM-ESTRO-ESUR-ISUP-SIOG. We comprehensively examine immunotherapeutic strategies currently available or under investigation for prostate cancer, with particular emphasis on ICIs, ACT with a focus on CAR T-cell therapy, combination approaches and therapeutic synergies, and predictive biomarkers of immunotherapy response. Additionally, we discuss the challenges and future directions in the implementation of immunotherapy for the management of prostate cancer.

## Simple Summary

Prostate cancer is the leading cause of cancer-related deaths worldwide and the second most common cancer in men. Immunological therapies include dendritic cell vaccines, immune checkpoint inhibitors, and adoptive cell therapy. This review explores the current landscape of prostate cancer. It analyzes treatment guidelines and examines immunotherapeutic strategies in use or research. Key areas include immune checkpoint inhibitors, adoptive cell therapy with CAR T-cell therapy, combination approaches, therapeutic synergies, and biomarkers that predict responses. Challenges and future directions in immunotherapy are also discussed.

## 1. Introduction

Prostate cancer (PCa) remains one of the most prevalent malignancies among men worldwide. In 2022, it was the second most frequently diagnosed cancer in males, with approximately 1.4 million new cases and nearly 397,000 deaths globally [1,2]. Despite its high incidence, prognosis is strongly stage-dependent: the five-year relative survival rate exceeds 95% for localized disease but declines sharply to nearly 30–35% in metastatic PCa [3], underscoring the critical importance of early detection and appropriate risk stratification.

The etiology of PCa is not fully understood; however, several well-established risk factors contribute to disease development. These factors are broadly classified as nonmodifiable—such as advancing age, ethnicity, family history, and inherited genetic mutations—and potentially modifiable, including obesity, metabolic syndrome, and smoking [4]. Age represents the strongest risk factor, with PCa incidence increasing markedly after 65 years [3]. A positive family history is associated with an elevated risk of aggressive disease, particularly when multiple first-degree relatives are affected or diagnoses occur at younger ages [4]. Heritability studies estimate that up to 57% of PCa susceptibility can be attributed to genetic factors [5]. In this context, germline mutations in DNA repair genes, including BRCA1/2, ATM, CHEK2, and mismatch repair (MMR) genes, have gained increasing relevance, especially in advanced disease, where such alterations are detected in approximately 10–20% of patients [4,6,7].

Clinically, most patients with localized PCa are asymptomatic, while lower urinary tract symptoms, erectile dysfunction, or hematuria typically emerge in more advanced stages. Bone is the most common site of metastasis, often resulting in pain or spinal cord compression [8]. Diagnosis relies on digital rectal examination, serum prostate-specific antigen (PSA) testing, and histological confirmation through prostate biopsy [9]. Current guidelines recommend the use of multiparametric magnetic resonance imaging to improve diagnostic accuracy and reduce unnecessary biopsies [8].

Tumor staging is performed using the TNM classification system (8th edition), applicable exclusively to histologically confirmed adenocarcinomas, while tumor aggressiveness is primarily assessed using the Gleason grading system according to the updated International Society of Urological Pathology (ISUP) criteria [8,10]. These classification tools are essential for prognostic assessment and therapeutic decision-making.

Treatment strategies for PCa are highly individualized and depend on disease stage, tumor biology, patient comorbidities, life expectancy, and patient preference [9,11]. Available management options include active surveillance, watchful waiting, radical prostatectomy, radiotherapy (RT), androgen deprivation therapy (ADT), and chemotherapy [9]. Active surveillance (AS) is the preferred approach for patients with low-risk localized disease and favorable life expectancy, whereas surgery and radiotherapy remain cornerstone curative treatments for localized and locally advanced PCa [8,11]. ADT constitutes the backbone of systemic treatment in advanced and metastatic disease; however, progression to castration-resistant prostate cancer represents a major clinical challenge, requiring multimodal therapeutic strategies, including chemotherapy and biomarker-driven targeted approaches [8,11].

Overall, optimal management of prostate cancer requires early diagnosis, accurate risk stratification, and personalized treatment selection to improve oncological outcomes and preserve quality of life.

## 2. Immunotherapy in Prostate Cancer

**Figure 1:**
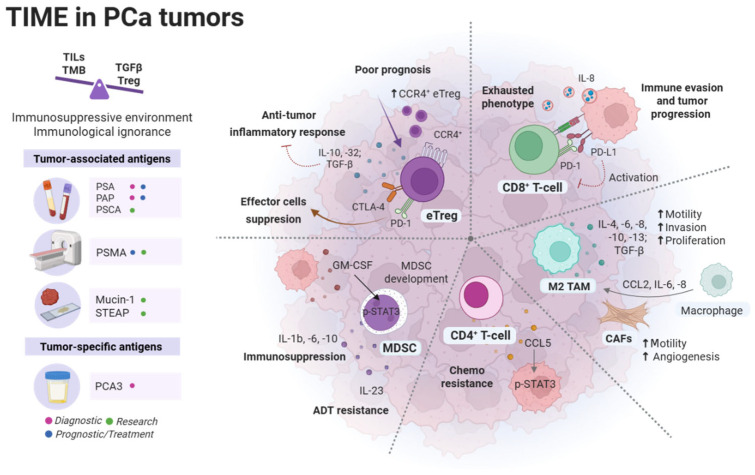
PCa tumor immune microenvironment (TIME). An immunosuppressive environment and Immunological ignorance characterize TIME in PCa tumors; some of the most relevant immune cells are effector Treg cells (eTreg), CD8^+^ T cells, M2 tumor-associated macrophages (TAMs), CD4^+^ T-cells, and myeloid-derived suppressor cells (MDSCs). Tumor-associated antigens and tumor-specific antigens can be used as diagnostic, prognostic/treatment, or research tools in PCa. PSA: Prostate-specific antigen; PSMA: Prostate-specific membrane antigen; PAP: Prostate acid phosphatase; PSCA: Prostate stem cell antigen; STEAP: Six-transmembrane epithelial antigens of the prostate; PCA3: Prostate cancer antigen 3.

PCa is classified as a “cold” tumor characterized by an immunosuppressive environment as a consequence of low tumor mutational burden (TMB), low levels of tumor-infiltrating lymphocytes (TILs), high transforming growth factor β (TGF-β) levels and regulatory T cells (Treg), and the expression of immune checkpoint molecules [12,13,14]. In fact, an RNA-seq data analysis of prostate adenocarcinoma from The Cancer Genome Atlas (TCGA) revealed that most PCa tumors (89.77%) exhibit “immunological ignorance”, characterized by low expression of genes involved in antigen processing and presentation, immune cell recruitment, and immune activation [15]. Some of the most abundant cells and components of PCa tumor immune microenvironment (TIME) are described in the following sections and summarized in Figure 1.

### 2.1. Tumor-Associated Macrophages (TAMs)

Tumor-associated macrophages (TAMs), specifically the CD68^+^ subtype, are the most abundant immune cells in the PCa tumor TIME. These macrophages exhibit two main phenotypes: M1-Type (anti-tumorigenic) that promotes a proinflammatory environment by releasing IL-1β, IL-12, IFN-γ, and TNF-α, and the M2-type (pro-tumorigenic), associated with tumor growth and metastasis [16,17]. They promote an immunosuppressive environment through the secretion of IL-13, IL-10, IL-8, IL-6, IL-4, and TGF-β [16,17]. In the PCa TIME, M2 TAMs are more numerous than M1 TAMs. Generally, a higher density of total TAMs in PCa correlated with worse prognoses and PSA failure [16,18].

Cancer-associated fibroblasts (CAFs) are another key cell type within the TIME that promote macrophage differentiation into TAMs by secreting factors like CCL2, IL-6, and IL-8. Among these, CCL2 exhibits strong chemotactic activity, which aids in recruiting macrophages [17].

### 2.2. CD8^+^ T-Cells and CD4^+^ T-Cells

Despite the growing body of literature addressing the immunological landscape of prostate cancer, the role of tumor-infiltrating CD8^+^ T cells remains equivocal. While several studies associate increased CD8^+^ T-cell infiltration with improved clinical outcomes [19], others report no prognostic benefit or even link these cells to immune dysfunction and tumor progression [20,21]. Such discrepancies likely reflect differences in study design, disease stage, spatial localization within the tumor microenvironment, functional status of CD8^+^ T cells, and methodological heterogeneity in immune profiling. Collectively, these conflicting findings preclude robust conclusions regarding the true prognostic or predictive value of tumor-infiltrating CD8^+^ T cells in prostate cancer, underscoring the need for standardized, functionally informed analyses to clarify their biological and clinical relevance.

On the other hand, the role of CD4^+^ T cells in PCa progression has been investigated, with studies showing an increase in CD4^+^ T-cell infiltration into PCa tumors following Docetaxel chemotherapy. The same research suggests that CD4^+^ T cells might contribute to chemotherapy resistance in PCa through modulation of the CCL5/STAT3 signaling pathway [22].

### 2.3. Regulatory T-Cells (Tregs)

A comparative study of tumor cellular populations PCa patients revealed an association between a higher percentage of CD4^+^ Treg cells and an increased risk of death from PCa [23]. A higher count of Tregs in patients with PCa was associated with a significantly reduced recurrence-free survival, as demonstrated in other studies. Additionally, the master transcription factor FOXP3, which regulates Treg function, is present in more than 80% of PCa samples. Some studies indicate that a higher number of intratumoral FOXP3^+^ Tregs is linked to a more advanced tumor stage but not to Gleason score [24].

The immunosuppressive activity of regulatory T cells (Tregs) within the prostate cancer tumor microenvironment is mediated predominantly through non-cytolytic mechanisms. Rather than relying primarily on perforin- and granzyme-dependent cytotoxicity, Tregs exert their suppressive effects mainly via the secretion of immunosuppressive cytokines, including IL-10, IL-35, and TGF-β [25], which inhibit effector T-cell activation and promote immune tolerance. In addition, Tregs engage in cell–cell contact-dependent suppression through the expression of inhibitory receptors such as CTLA-4 and LAG-3, leading to impaired antigen-presenting cell function and reduced co-stimulatory signaling. Metabolic mechanisms further contribute to Treg-mediated immunosuppression, notably through CD25-dependent sequestration of IL-2 and the generation of adenosine via the CD39/CD73 pathway, resulting in metabolic deprivation and functional exhaustion of effector T cells. Additionally, Tregs contribute to cancer progression, stimulating immunosuppressive M2 macrophages [17,26]. Moreover, a high expression of the C-C chemokine receptor 4 (CCR4) in effector T regulatory cells (eTreg) has been associated with a poor prognosis and with a potential to progress to CRPC in patients with PCa due to the migration of more CCR4^+^ eTreg into the tumor [27]. Collectively, these mechanisms foster an immunosuppressive microenvironment that supports tumor progression and immune evasion in prostate cancer.

### 2.4. Myeloid-Derived Suppressor Cells (MDSCs)

Myeloid-derived suppressor cells (MDSCs) are a heterogeneous group of cells that can be classified as polymorphonuclear (PMN-MDSC CD14^+^) or monocytic (M-MDSC CD15^+^). MDSCs are abnormally activated neutrophils and monocytes that exhibit strong immunosuppressive functions [28]. In a mouse model of PCa, inhibiting pSTAT3 in MDSCs results in the prevention of tumor growth and spread. The authors found that PCa cells secrete GM-CSF, which promotes MDSC development through pSTAT3 [29]. In turn, an in vitro model using primary human monocytes cocultured with DU145, PC3, and LNCaP-IL6 PCa cell lines demonstrated that galiellalactone-mediated STAT3 inhibition can suppress the induction of primary human MDSC-like monocytes by PCa cells ex vivo [30]. Additionally, in the same study, coculturing monocytes with conditioned medium from PCa cells reduced the levels of immunosuppressive and tumor-promoting factors, such as IL-6, IL-1β, and IL-10, in monocytic MDSCs [30].

### 2.5. Tumor-Associated Antigens (TAAs) in Prostate Cancer

Tumor-associated antigens (TAAs), encoded by non-mutated genes but aberrantly expressed in tumor cells, are recognized by the immune system as altered self-antigens, thereby initiating a humoral immune response [31]. Antigen-presenting cells (APCs) internalize tumor material, process it, and present TAAs on their surface for T- and B-cell activation [32]. Among the principal biomarkers studied in prostate cancer is PSA, first characterized in seminal fluid by Hara et al. (1971) [33]; although PSA measurement in serum is highly sensitive, it lacks specificity for prostate cancer because elevated levels also occur with aging and benign conditions such as benign prostatic hyperplasia (BPH) and prostatitis [32,34].

### 2.6. Prostate-Specific Membrane Antigen (PSMA)

The prostate-specific membrane antigen (PSMA), also known as folate hydrolase 1, was described by Horoszewicz et al. (1983) [35] while establishing the LNCaP cell line from a metastatic lesion of human prostatic adenocarcinoma and was first cloned ten years later by Israeli, Powell, Fair, and Heston [36].

PSMA is a type II transmembrane glycoprotein consisting of 750 amino acids, featuring an intracellular domain, a transmembrane domain, and a large extracellular domain; within the extracellular domain, the binding domain forms a dimer that binds to glutamate and glutamate-like structures and functions as a glutamate carboxypeptidase [37,38].

PSMA is expressed in healthy and malignant prostate tissues, but in cancer cells it is upregulated [38,39,40]. Heston (1997) observed that as prostate cells turned cancerous, the expression ratio of the two message forms varied almost 100-fold, with the cytosolic form of PSMA being more common in normal cells and the membrane form more prevalent in cancer cells [41]. This glycoprotein is especially useful in PCa for the association of higher expression levels with disease progression, recurrence, invasion, and treatment resistance [38,42]. PSMA is used as TAA imaging and therapeutic target [39] theranostics (a combination of therapy and diagnostics) [38].

### 2.7. Prostate Acid Phosphatase (PAP)

PAP is a glycoprotein secreted by prostate epithelial cells [39] and discovered in the serum of men with metastatic PCa in elevated levels [43]. PAP is found in two forms: cellular PAP (cPAP) and secreted PAP (sPAP) [44]. Before the widespread use of PSA, PAP was used as a biomarker for PCa detection, but due to its nonspecific expression in multiple organs, PSA replaced it [44]. Despite that, PAP has a robust and persistent expression in immunohistochemical staining of bone metastasis derived from mCRPC [45,46], contrary to PSA and AR expression in the same tissue, in which the expression is limited and modest, respectively [47].

### 2.8. Prostate Stem Cell Antigen (PSCA)

Prostate stem cell antigen (PSCA) is a glycosylphosphatidylinositol (GPI)-anchored cell surface protein, a member of the Thy-1/Ly-6 family. PSCA participates in cellular signaling, proliferation, cell cycle regulation, and immune responses [48]. PSCA mRNA is expressed in normal basal and secretory cells of the prostate and is highly upregulated in progression to androgen-independent PCa tumors, in higher Gleason scores, and in advanced tumor stages [48,49,50]. Recent studies indicate that microRNA-34a, a potent tumor-suppressive miRNA, functions as a key regulator of cancer stem cell properties by targeting multiple molecules involved in cell survival [51] and may be particularly effective in prostate tumors associated with TP53 mutations [52].

In situ hybridization (ISH) and immunohistochemistry (IHC) analysis have shown that PSCA mRNA and PSCA protein are weakly expressed or not expressed in BPH and low-grade prostatic intraepithelial neoplasia (PIN). In contrast, in high-grade prostatic intraepithelial neoplasia (HGPIN), expression is higher. Additionally, in PCa, higher protein and mRNA levels of PSCA correlate with tumor grade, stage, and progression toward androgen independence [53].

### 2.9. Mucin-1

Mucin1 (MUC1) is a heterodimeric transmembrane protein, a member of the mucin family of anti-adhesion molecules [54,55]. Mucin1 is mainly found on the apical surface of most glandular epithelial cells throughout the human body [55]. Abnormal MUC1 expression and glycosylation in cancer activate multiple pathways, promoting tumor migration, invasion, and faster growth [55].

MUC1 is formed by two subunits: an N-terminal highly glycosylated (MUC1-N) and an oncogenic C-terminal transmembrane (MUC1-C), which is overexpressed in CRPC and in Neuroendocrine PCa (NEPC) [56]. In addition, in PCa, overexpression of MUC-1 in tissue has been correlated to higher Gleason grades and more advanced pathologic stage [57].

### 2.10. Six-Transmembrane Epithelial Antigens of the Prostate (STEAP)

The six-transmembrane epithelial antigens of the prostate (STEAP1, STEAP2, STEAP3, and STEAP4) form a family of metalloproteinases that play roles in iron and copper regulation. They also participate in diverse cellular functions, such as molecular trafficking within the endocytic and exocytic pathways, as well as the regulation of cell proliferation and apoptosis [58]. STEAP proteins are seen as potential biomarkers across various cancers like prostate, pancreas, colon, and ovary, playing roles in tumor initiation and progression. Evidence suggests that these proteins are abnormally expressed in different cancer types, driving tumor cell growth, movement, and malignant transformation [59,60].

In PCa, STEAPs are recognized as potential biomarkers and as therapeutic targets: STEAP1 and STEAP2 expression on the cell’s surface is elevated in prostate neoplasia and is strongly correlated with more aggressive PCa phenotypes and STEAP1 with poorer prognosis; conversely, STEAP3 has shown tumor suppressor activity by promoting apoptosis through a caspase-3 dependent mechanism; dysregulation of STEAP4 is associated with tumor grade, response to treatment, and decreased overall survival in PCa patients [59].

### 2.11. Prostate Cancer Antigen 3 (PCA3)

Prostate cancer antigen 3 (PCA3), initially known as differential display code 3 (DD3), is a long noncoding RNA (LncRNA). PCA3 is involved in PCa cell survival by modulating AR signaling [61]. PCA3 is especially valuable due to its specific expression in normal prostate tissue and its overexpression in more than 94% of primary PCa [61,62].

PCA3 is not expressed in other prostate pathologies besides PCa; furthermore, it can be detected in a minimally invasive manner through a urinary sample. Its quantification is recommended to be used in combination with other tumor biomarkers such as PSA [38,63,64].

### 2.12. Immune Evasion Mechanisms Employed by Prostate Tumor Cells

In general, the immune response to cancer begins with the release of antigens from cancer cells, followed by antigen presentation, priming and activation of T-cells, their transfer and infiltration into the tumor, specific recognition, and ultimately tumor cell elimination [14].

The immune response to PCa cells starts with cancer cells releasing antigens, which are then detected by APCs such as dendritic cells (DCs), followed by antigen presentation and activation by major histocompatibility complex I and II (MHC-I/II) to T cell receptor (TCR) of CD8^+^ and CD4^+^ T cells, respectively [14]. After the TCR-activating signal is transduced, a co-stimulatory signal from the CD28 co-receptor must be received before correct T-cell activation [65].

The central cells effective in eliminating neoplastic cells are cytotoxic T cells (CTLs), which use two main mechanisms: perforin and granzyme-dependent pathways. Perforin inserts into the cancer cell membrane, forming pores that facilitate the entry of granzymes to activate the apoptotic pathway. The second involves death receptor signaling by FasL-Fas and TRAIL-DR5 interactions. Another indirect way in which CTLs eliminate tumor cells is by releasing proinflammatory cytokines like IFN-γ and TNF-α, both of which have antiproliferative effects on tumor cells and contribute to TME immune remodeling [65,66].

The immunosuppressive environment in PCa tumors can be explained by four mechanisms proposed by López-Campos et al.: (1) lower density of cytotoxic cells like CD8^+^ T lymphocytes and natural killer (NK), in contrast to a higher presence of Treg cells, CD4^+^ T lymphocytes, and M2 macrophages. (2) Exhausting cytotoxic and dendritic cell phenotype by high PD-1 and CTLA-4 overexpression. (3) Immune-suppressive cytokines produced by CD4^+^ T lymphocytes and M2 macrophages as IL-10, IL-23, TGF-β, CCL2, and CCL22; these cytokines also promote tumor progression and metastasis. (4) Intratumoral molecules like decoy receptor 3 (Dcr3) promote tumor growth by inhibiting TNFR [13].

## 3. Immunotherapy Landscape in Prostate Cancer

### 3.1. Early Attempts at Immunotherapy in Prostate Cancer: Early Cell Vaccines and Other Approaches

Tumor-specific active immunotherapy, particularly in the form of tumor vaccines, emerged as a strategy to stimulate the immune system. The goal of these vaccines is to maintain long-term anti-tumor responses, thereby mitigating the risks of cancer recurrence and metastasis [67]. Formulating PCa vaccines involves isolating and extracting or synthesizing a TSA or tumor-related antigen; tumor vaccines can be based, primarily, on dendritic cells, antigen, nucleic acid, and tumor cells [67].

Initial efforts to develop immunological treatments for PCa faced limitations due to poor lymphocyte infiltration and, generally, an immunosuppressive tumor environment. However, there are now several immunological therapeutic strategies available for PCa, with the main ones being dendritic cell-based vaccines, immune checkpoint inhibitors (ICIs), and adoptive cell therapy [68]. The first immunologically approved treatment for PCa was the dendritic cell-based vaccine Sipuleucel-T (Provenge), used for treating asymptomatic or minimally symptomatic mCRPC [69,70].

Although Sipuleucel-T early trials in metastatic PCa indicated a survival benefit, they did not show clear effects on disease progression [71,72]. To confirm the vaccine’s efficacy, the phase III trial NCT00065442 [73], called the Immunotherapy for Prostate Adenocarcinoma Treatment (IMPACT) trial, was designed with overall survival as the primary endpoint. IMPACT was a double-blind, placebo-controlled, multicenter study involving 512 men with mCRPC; 341 patients received Sipuleucel-T and 171 received a placebo, with infusions administered every 2 weeks for a total of three doses. The Sipuleucel-T group showed a 22% relative reduction in the risk of death (hazard ratio [HR] = 0.78; 95% confidence interval [CI] = 0.61 to 0.98; *p* = 0.03), which translated to a 4.1-month increase in median survival (25.8 months in the Sipuleucel-T group vs. 21.7 months in the placebo group); however, no statistically significant effect in progression-free survival (PFS) in the vaccine group was observed (3.7 months in the Sipuleucel-T group vs. 3.6 months in the placebo group. HR = 0.95; 95% CI, 0.77 to 1.17; *p* = 0.63) [69].

Currently, the use of the Sipuleucel-T vaccine is limited due to its modest effectiveness against mCRPC as monotherapy and the high costs associated with treatment, which are often prohibitive for many patients. However, the immunologic benefits seen in combination therapy settings provide a rationale for future large-scale clinical trials [68].

In 2017, the FDA approved pembrolizumab (KEYTRUDA, Merck & Co., Rahway, NJ, USA), a PD-1 immune checkpoint inhibitor (ICI), for treating adults and children with unresectable or metastatic mismatch repair deficiency (dMMR) or high microsatellite instability (MSI-H) solid tumors. This includes cases that have progressed after previous treatments and lack better options [74].

Recent research has concentrated on combination strategies that aim to convert the tumor from an immune-cold to an immune-hot state by use of combined treatment, for example, high dose-rate brachytherapy (HDRBT) [75], chemotherapy or hormone therapy, enhancing in this way the vulnerability of the tumor to immune attacks [76].

### 3.2. Lessons Learned from Previous Studies and Their Influence on Current Strategies

The initial attempts at immunological treatments for PCa highlighted the importance of effective antigen presentation, the possible necessity for immunostimulatory adjuvants, and the PCa tumor’s ability to evade immune responses [76].

Emerging perspectives from previous immunotherapy trials suggest that successful outcomes in PCa do not necessarily require complete disease eradication. Current approaches are redefining treatment success by focusing on establishing sustained immune-mediated control that keeps the cancer in a dormant, slow-growing state rather than aiming for total elimination [77]. This shift highlights an important lesson: turning an aggressive disease into a manageable, chronic condition through immune system modulation may be a more feasible and clinically meaningful approach. Along with ongoing efforts to identify predictive biomarkers and optimize combination treatments, this strategy offers an encouraging new direction that has influenced current immunotherapy approaches.

## 4. Immune Checkpoint Inhibitors in Prostate Cancer

### 4.1. Explanation of Immune Checkpoint Inhibitors and Their Mechanisms of Action

Immune checkpoint inhibitors mark a significant step in cancer therapy. Checkpoint molecules such as PD-1 and CTLA-4 are part of the immune system’s intrinsic mechanisms designed to prevent excessive activation and the development of autoimmunity. Although these molecules serve protective roles, cancer cells can exploit these pathways to suppress immune responses against tumors. The development of monoclonal antibodies that target these checkpoints has shown that releasing T cells from inhibitory signals can restore effective tumor immune surveillance. This approach has improved outcomes for previously hard-to-treat cancers and established immunotherapy as a core strategy in modern oncology [78].

In a normal context, PD-1 binding with its ligands PD-L1 or PD-L2 sends inhibitory signals to T lymphocytes, promoting tolerance and maintaining immune homeostasis while decreasing the risk of autoimmune diseases [79]. The role of PD-1/PD-L1 varies with the physiological context: although it helps sustain immune tolerance, in cancer settings, it can diminish the immune response against tumor cells [80]. PD-1 is expressed on T cells during thymic development when they are active and tumor-specific. It is also found on macrophages, B lymphocytes, some dendritic cells, monocytes, myeloid cells, and NK cells [81,82].

### 4.2. Review of Clinical Trials and Outcomes with Immune Checkpoint Inhibitors in Prostate Cancer, Including Survival and Response Data

To examine the current landscape of immune checkpoint inhibitors in prostate cancer, a search was conducted on ClinicalTrials.gov (https://clinicaltrials.gov/) of the National Library of Medicine using the following criteria—Condition/disease: Prostate Cancer; Other terms: immune checkpoint inhibitors; Intervention/treatment: Immune checkpoint inhibitors, atezolizumab. Clinical trials with an unknown (because the study has passed its completion date and the status has not been verified in more than two years), withdrawn, or suspended without results status were not considered. The clinical trials resulting from this are listed in Table 1. The studies with published results at the time of this review are NCT03406858 [83], NCT03016312 [84], and NCT04446117 [85].

NCT03406858 is a phase II trial that investigated the effectiveness of pembrolizumab and HER2 bispecific antibody (bsAb)-armed activated T cells in treating patients with mCRPC. Their results showed grade 1–2 infusion-related adverse events. The primary endpoint of 6-month PFS was met in 5 out of 14 patients, with a median PFS of 5 months. The median overall survival was 31.6 months [86].

NCT03016312 is a phase III trial that assessed the safety and effectiveness of combining the ICI atezolizumab with enzalutamide, compared to enzalutamide alone, in patients with mCRPC after previous treatment failure. The results did not achieve the primary endpoint of improving overall survival in unselected patients (HR = 1.12; 95% CI, 0.91–1.37; *p* = 0.28) [87].

NCT04446117 is a phase III trial designed to evaluate the safety and efficacy of cabozantinib combined with the ICI atezolizumab vs. a second novel hormonal therapy in mCRPC patients who have previously received only one novel hormonal therapy. After a median follow-up of 11.8 months, cabozantinib plus atezolizumab significantly improved PFS compared to ARPI switch (median 6.3 months vs. 4.2 months; HR = 0.65, 95% CI: 0.50–0.84; *p* = 0.0007). The study also reported a 56% incidence of grade 3–4 adverse events in 284 patients treated with cabozantinib plus atezolizumab and 26% in patients who switched to ARPI. Serious adverse events thought to be related to treatment occurred in 16% of patients in the cabozantinib plus atezolizumab group and in 4% of patients in the ARPI switch group [88].

Furthermore, to strengthen the ICIs clinical trials results in Table 1, other studies focused specifically on bispecific antibodies (bsAbs) were identified with the following criteria: Condition/disease: Prostate Cancer; Intervention/treatment: Bispecific antibodies, Bispecific antibodies \(bsAB\) listed in Table 2. NCT04740034 [89] is a phase I study evaluating the safety, clinical pharmacology, and clinical activity of AMG 340, a PSMA × CD3 T-cell engaging bispecific antibody, in mCRPC patients who have received two or more prior lines of therapy. The trial is listed as terminated by the sponsor’s strategic decision, with results on ClinicalTrials.gov, but published results were not provided. NCT04424641 [90] was a phase I dose-escalation trial evaluating the safety of GEN1044 (CD3x5T4 bispecific antibody) in patients with malignant solid tumors, including prostate cancer. The study was completed after the maximum tolerated dose was established. Published results are pending.

### 4.3. Analysis of the Efficacy of These Inhibitors in Different Disease Stages

Although PCa tumors are generally considered immunologically cold due to their poor immune response and lower immunological cell infiltration, there are apparent differences between the different stages of PCa; while early PCa stages show a predominantly cold TIME, the more aggressive stages such as CRPC may exhibit increased TILs, possibly in response to changes in the TIME caused by conventional treatments; therefore, their immunogenicity may become more responsive to immunological treatment approaches [76].

## 5. Adoptive Cell Therapies for Prostate Cancer

Adoptive cell therapies (ACT) for prostate cancer represent an emerging field aimed at overcoming the limitations of conventional treatments in advanced disease, particularly mCRPC [91]. These therapies encompass several modalities, including chimeric antigen receptor (CAR) T cells, TCR-engineered cells, NK cell approaches, and TIL therapies [92]. Adoptive cell therapy for cancer involves modifying a patient’s immune cells, primary T cells, to target and destroy tumor cells. This is achieved by genetically engineering immune cells to express receptors that recognize specific proteins on cancer cells, effectively turning them into professional “killer” cells that can fight the disease.

Beyond CAR-T cells, other ACT modalities, such as TCR-engineered T cells, NK cell therapies, and TIL therapies, are being explored; however, clinical data in prostate cancer remain relatively preliminary, and most clinical investigations to date have primarily focused on CAR-T cell strategies [92].

CAR T-cell therapy is an advanced form of adoptive cell immunotherapy in which autologous T Lymphocytes are genetically engineered to express a synthetic receptor that targets tumor-associated antigens independently of MHC presentation [37]. The process involves isolating T cells from the patient’s peripheral blood, followed by transduction with a viral vector encoding the CAR construct, which is typically composed of an extracellular antigen-binding domain derived from a monoclonal antibody, a transmembrane domain, and one or more intracellular signaling domains [37]. These modified T cells are expanded ex vivo and subsequently infused into the patient, where they mediate targeted cytotoxicity against cancer cells expressing the specific antigen. CAR-T cell immunotherapy (chimeric antigen receptor T-cell therapy) was considered an exciting new approach to treat some patients with very advanced forms of cancer.

CAR T-cell therapy has demonstrated significant clinical efficacy in certain hematologic malignancies, and it is currently under active investigation for its application in solid tumors. It promises a potential treatment for prostate cancer, particularly in advanced and metastatic stages; however, it is still in its early stages of development.

In the context of prostate cancer, CAR-T cells are designed to target specific antigens such as PSMA and PSCA, which are abundantly expressed on malignant prostate cells [93]. PSMA is highly expressed on prostate cancer cells, especially in advanced and castration-resistant disease [37]. PSMA is not only abundant in malignant prostate cells but also localizes to the tumor neovasculature, thereby doubling as an effective target to disrupt the tumor’s supportive microenvironment [37].

**Figure 2:**
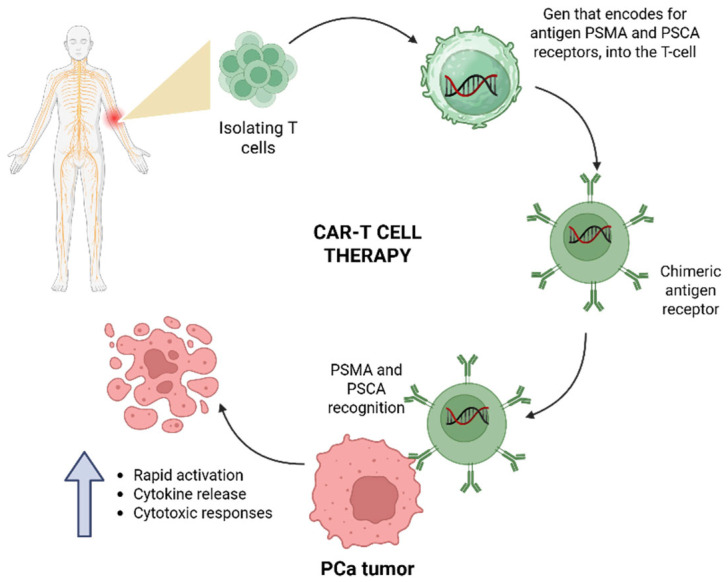
CART T-cell therapy in PCa tumors. Isolating T cells from the patient’s peripheral blood, followed by transduction with a viral vector encoding the CAR construct designed to target specific antigens such as PSMA and PSCA, which are abundantly expressed on malignant prostate cells.

Another important antigen targeted by CAR T cells in prostate cancer is PSCA. PSCA expression is correlated with higher Gleason scores, tumor progression, and metastatic potential, making it an attractive candidate for immunotherapeutic intervention [37]. Clinical trials, such as (NCT03873805) [94] and (NCT05805371) [95], specifically evaluate CAR T cells that target PSCA, capitalizing on its restricted expression in malignant prostate tissue. The general process of CAR T-cell therapy development and application is described in Figure 2.

In addition to PSMA and PSCA, a subset of studies has investigated other cell surface antigens such as EpCAM, a protein frequently found on cancer cells that has shown promise in preclinical and clinical studies for treating solid tumors [96]. For instance, the clinical trial employs CAR T cells directed against EpCAM, a broadly expressed epithelial antigen seen in various cancers, including prostate cancer, but its expression in multiple tissues limits its prostate specificity compared to PSMA or PSCA (NCT03013712) [97] (NCT02915445) [98].

A common theme across these approaches is the focus on membrane-bound antigens that are accessible to the immune synapse of CAR T cells. This strategy ensures that engineered T cells, upon encountering their target antigen on tumor cells, undergo rapid activation, cytokine release, and subsequent cytotoxic responses [99].

Preclinical and early clinical data have demonstrated that CAR T cells targeting PSMA can effectively recognize and eliminate tumor cells in vitro and xenograft models; however, challenges such as the immunosuppressive microenvironment and suboptimal CAR T cell persistence remain to be overcome [37,93].

The use of CAR T cells against these antigens, primarily PSMA and PSCA, exemplifies a broader trend in immunotherapy toward the use of highly specific cell-surface markers that allow for the targeted destruction of tumor cells while minimizing damage to normal tissue [100].

Developing effective cell therapies for prostate cancer is extremely challenging due to the unique immunobiology of this malignancy and its highly immunosuppressive TME [101]. A significant barrier is the natural immune tolerance to self-antigens expressed by prostate cancer cells because these antigens are not recognized as foreign, and strategies such as xenogeneic modification or dendritic cell priming are required to break tolerance and generate a robust anti-tumor immune response [101] (NCT00105053) [102].

The immunosuppressive TME is characterized by inhibitory cytokines such as TGF-β and interleukin-10 (IL-10) and by recruiting regulatory T cells, myeloid-derived suppressor cells, and tumor-associated macrophages that collectively dampen the activity and persistence of infused therapeutic cells, such as CAR-T cells [101,103].

Another inherent challenge for solid tumors is the physical barriers, including a dense extracellular matrix and aberrant vasculature, which limit the infiltration of TILs and thereby reduce their potential for effectively eradicating tumor cells [99].

Tumor antigen heterogeneity represents an additional significant challenge due to the variable expression of antigens such as PSMA, PSCA, and EpCAM, and this variability not only complicates the choice of target but also increases the risk of antigen-loss variants emerging during therapy [99,104].

PCa exhibits a low production of DAMPs, which affects the activation of DCs, subsequently reducing the production of immune-related chemokines and cytokines, thereby contributing to tumor progression. The TME additionally forms a physical barrier that restricts efficient T-cell trafficking and infiltration into tumor sites, contributing to the classification of prostate cancer as an immunologically “cold” tumor with limited immune cell infiltration, or with an increased presence of T regulatory and T helper 17 phenotypes [105]. Even when TILs are present, their activation is often compromised; many of these cells exhibit functional exhaustion or even regulatory characteristics that limit their capacity to mount effective anti-tumor responses [106,107].

Adoptive cell therapies for prostate cancer are rapidly evolving with advanced genetic modifications and combinatorial regimens that aim to overcome the intrinsic barriers posed by the immunosuppressive TME. Although most clinical efforts have focused on CAR-T cell approaches targeting antigens such as PSMA, PSCA, and STEAP1, ongoing trials and preclinical investigations are expanding the scope to include alternative ACT strategies for advanced prostate cancer to overcome the highly immunosuppressive microenvironment [91,108].

To examine the current landscape of clinical trials of adoptive cell therapies in PCa, a search was conducted on ClinicalTrials.gov (https://clinicaltrials.gov/) of the National Library of Medicine using the following criteria: Condition/disease: Prostate Cancer; Other terms: Adoptive Cell Therapies. Clinical trials with an unknown (because the study has passed its completion date and the status has not been verified in more than two years), withdrawn, suspended without results, or not yet recruiting status were not considered. Additionally, other studies (NCT03873805 [94], NCT05805371 [95], NCT00105053 [102]) that the criteria did not show, but we considered important, are included in Table 3.

The ongoing, non-recruiting phase I trial NCT03873805 [94] investigates the side effects and optimal dose of PSCA-chimeric antigen receptor (CAR) T cells for treating patients with prostate stem cell antigen-positive (PSCA+) mCRPC. The results in the 14 involved patients demonstrated acceptable safety with one DLT (grade 3 cystitis) and grade 1–2 cytokine release syndrome in 5/14 patients. Clinical responses included PSA declines >30% in 4/14 patients and radiographic improvements, though CAR T cell persistence was limited to <28 days post-infusion [93].

## 6. Combination Approaches and Synergies

A critical evaluation of negative, inconclusive, and prematurely terminated immunotherapy trials in prostate cancer highlights the central role of tumor-specific immunobiology in shaping clinical outcomes. Unlike immunotherapy-responsive malignancies, prostate cancer is characterized by a low tumor mutational burden, resulting in limited neoantigen generation and weak baseline immunogenicity [12,13,14]. This feature is compounded by low densities of functional tumor-infiltrating lymphocytes, with CD8^+^ T cells often exhibiting an exhausted or dysfunctional phenotype rather than effective cytotoxic activity [19,20,21].

Moreover, the prostate cancer tumor microenvironment is dominated by an immunosuppressive myeloid compartment, including M2-polarized tumor-associated macrophages and myeloid-derived suppressor cells, which actively inhibit T-cell priming, trafficking, and effector function through cytokine secretion (e.g., IL-10, TGF-β), metabolic competition, and immune checkpoint engagement [16,17,18,25,26,109,110,111]. These biological constraints provide a mechanistic explanation for the modest efficacy observed in multiple immune checkpoint inhibitor trials, including those that failed to demonstrate survival benefit in unselected patient populations or were terminated due to limited clinical activity [87,88,112].

Collectively, these findings underscore that resistance to immunotherapy in prostate cancer is not solely attributable to therapeutic design but is deeply rooted in tumor-intrinsic and microenvironmental features. This recognition supports the rationale for combination strategies aimed at remodeling the tumor immune microenvironment, enhancing antigenicity, and selectively enriching for immunologically responsive patient subsets.

Proliferative response to antigen-specific T-cell stimulation, T-cell infiltration into the prostate, and faster lymphocyte recovery following chemotherapy have been linked to ADT [28,113]. Under androgen deprivation therapy, a cohort of 20 patients showed an increase in the naïve CD4 subpopulation (CD45RO^−^/CCR7^+^) in peripheral blood after treatment. The other subpopulations were produced relatively regularly over time. This suggests that ADT not only affects the frequency of T lymphocyte subsets but also their responsiveness to routine costimulatory molecules [114]. In the same way, other researchers have also described how ADT induces the infiltration of CD4^+^ and CD8^+^ T lymphocytes in prostate cancer compared with healthy prostate tissue, which could be detected during the first month of treatment [115].

In 2021, a study reported that androgen receptor signaling has a potential effect on myeloid cell metabolism and function enhancing tumor-promoting capability in prostate cancer models. They concluded that androgen receptor blockade might inhibit prostate tumor growth; however, it also promoted tumor resistance by enhancing the immunosuppressive activity of myeloid cells [116].

In patients with metastatic castration-resistant prostate cancer treated with Enzalutamide or Abiraterone, decreased levels of fibroblast growth factor, granulocyte macrophage colony-stimulating factor (GM-CSF), IL-10, and IL-6 have been demonstrated in patients with androgen deprivation compared to de novo castration-resistant patients [117].

The immunologic impact of enzalutamide was recently demonstrated in patients with non-metastatic prostate cancer sensitive to hormone therapy. The study included 38 patients treated with Enzalutamide and found increased levels of PSA-targeted antigen-specific T cells and NK cells, as well as decreased MDSCs in the blood. Finally, no association was demonstrated between clinical response and immunologic changes [118].

As previously mentioned, chemotherapy is used in cases of metastatic castration-resistant prostate cancer. After chemotherapy management, some changes in the immune response have been demonstrated. Docetaxel regulates several pathways related to the immune system. T cells (IFNγ and TNFα gene sets), B cells, and NK mediators of immunity are strengthened after diocetaxel therapy with increases in CD8^+^, CD3^+^, and CD4^+^ [22]. Preclinical studies have shown that chemoresistance to Docetaxel is mediated by the cytokine CCL5 secreted by CD4^+^, which increases the aggressive potential of stem cell populations of prostate cancer. Also, this could potentially activate the PI3K/Akt and STAT3 pathways, which could be related to aggressiveness and cell migration [22].

The metronomic chemotherapy (MCT) approach involves the continuous and low-dose administration of chemotherapeutic agents [119]. This method significantly reduces side effects and improves disease management by modulating the immune system through multiple mechanisms: it triggers immunogenic death of cancer cells, selectively reduces regulatory Tregs, enhances antigen presentation by activating DC function and enhancing tumor cell immunogenicity, suppresses MDSCs, and stimulates immune effector cells such as tumor-specific T cells and γδT cells [119,120].

Implementing MCT in mCRPC demands context-specific considerations: in advanced disease stages where therapeutic options are exhausted and trial participation is impractical, especially given recent negative results with immunotherapy monotherapy; in geriatric or vulnerable patients who need gentler alternatives to aggressive biweekly or triweekly cytotoxic regimens; and in healthcare settings in developing regions where financial barriers hinder access to high-cost modern pharmaceuticals [119].

The effect of RT begins with a proinflammatory cascade that increases the systemic response to immunotherapy. High doses of RT stimulate proinflammatory chemotherapies such as TNFα, IL-1, IL-6, and IL-8, increasing the presence of T cells, upregulating MHC class I molecules that facilitate antigen presentation and the presence of effector cells through the production of chemotactic chemokines, and upregulating vascular adhesion molecules [121].

The effects of high-dose brachytherapy on the immune response in patients with prostate cancer have been studied. Several molecules were upregulated in response to radiation, improving the response to immunotherapy [75].

Overall, only 3–5% of prostate cancer patients respond to PD-1/PD-11 blockade [122]. However, it has been reported that patients with MSI, approximately 3% of all prostate cancer patients, are associated with a high mutation rate and subsequently low immune checkpoint inhibitors. These data suggest positive approval for patients with MSI cancer. These attractive results suggested that in some of these patients, the MSI phenotype might be acquired by dynamic changes in TME and response to previous therapies [123].

Accumulating studies confirm that hypoxia potently induces HIF-1α-dependent PD-L1 expression on tumor cells, suggesting that PD-L1 expression may be upregulated in hypoxic tumor cells to promote immune escape of cytotoxic T cells. It has been suggested that co-blocking PD-L1 and HIF-1α signaling could represent a promising approach to enhance cytotoxic T cell activity [124].

At this point in the timeline, a significant number of articles can be found on the effects of immunotherapy on prostate cancer. Scientific evidence has already been applied to the clinical field for malignant neoplasia of the genitourinary tract, especially for bladder and kidney cancer. However, international clinical practice guidelines still do not include immunotherapy as a treatment for patients with prostate cancer at any stage. However, the large amount of research being conducted is just on the cusp of reaching a turning point in the prostate cancer pathway so that it can be implemented.

Overall, the immunomodulatory potential of metronomic chemotherapy in prostate cancer is supported mainly by preclinical and translational studies, while prospective clinical validation remains limited. Although low-dose chemotherapy has been shown to favorably modulate the tumor immune microenvironment, its clinical relevance as an immunotherapeutic strategy has yet to be firmly established. Consequently, metronomic chemotherapy is best considered a complementary approach whose true value will depend on well-designed combination trials incorporating immune-based endpoints and biomarker-guided patient selection.

## 7. Predictive Biomarkers of Response to Immunotherapy in Prostate Cancer

### 7.1. General Description of Biomarkers That Predict Response to Immunotherapy

The clinical development of ICIs has been used in a new era of anti-tumor therapy with a significant survival advantage observed in multiple diseases [125]. Predictive biomarkers are measurable biological factors that help determine the probability of a patient’s response to a specific treatment. These biomarkers are crucial for identifying patients who are most likely to benefit from immune-based treatments, such as immune checkpoint inhibitors [126], cancer vaccines [127], and adoptive T-cell therapies [128]. With the development and continuous improvement of multiplex immunohistochemical technology, high throughput sequencing, and microarray technology, a variety of biomarker strategies have emerged and gradually realized the process from the identification of a single marker to the development of multifactorial synergistic predictive markers.

Monitoring efficacy and disease development, guiding clinical trial design, as well as a further understanding of drug resistance mechanisms and tumor prognosis is crucial [129]. Furthermore, we deeply analyze the exploration and research progress of predictive biomarkers such as PDL-1 expression, TMB, MSI, and other genomic markers to identify effective tools against PCa.

### 7.2. Discussion of Specific Biomarkers Relevant to Prostate Cancer, Such as PDL-1 Expression, TMB, MSI, and Other Genomic Markers

In recent years, immunotherapy against cancer has yielded encouraging results and significantly changed the treatment landscape, owing to its efficacy and minimal side effects [130]. PD-1, also known as cluster of differentiation 279 (CD279), is encoded by the PDCD1 gene located on chromosome 2q37.2 PD-1 is widely expressed in a range of immune cells, such as T cells and dendritic cells [131]. High expression of PD-1/PD-L1 is associated with the clinical features of prostate cancer [132]. Compared with normal prostate epithelium, the PD-1 promoter is significantly hypermethylated in TILs, and PD-1 methylation is negatively related to the expression of PD-1 mRNA in prostate cancer [133].

Significant correlations between high TMB and response to ICIs have been reported in several cancer types [134], including urothelial carcinoma [135], small cell lung cancer (SCLC) [136], prostate cancer [137], and others.

### 7.3. Evaluation of the Challenge and Potential Solutions for Identifying Reliable Predictive Biomarkers

Personalized medicine relies upon individualized diagnosis that provides molecular information delineating optimal therapeutic strategies. For many diseases, but especially cancer, the development of predictive biomarkers requires performing assays directly on the diseased tissue or tumor. Over the past decade, there has been a rapid expansion in the identification of prognostic and predictive biomarkers within the research field; however, only a limited number have been successfully translated into routine clinical practice [138]. In contrast, imaging tools such as computed tomography (CT) and positron emission tomography (PET) are already playing a significant role in cancer prevention, as well as in the early detection and clinical management of patients with cancer [139].

At present, AI-based computational frameworks combine clinical and genomic data to advance precision medicine strategies, minimizing trial-and-error therapeutic decisions and leading to improved patient outcomes [140]. Machine learning has demonstrated considerable promise in enhancing the diagnosis, prognostic assessment, and therapeutic planning of prostate cancer (PCa); however, a persistent translational gap remains between the development of computational algorithms and their adoption in clinical practice [141].

One of the goals of personalizing medicine is to use the growing understanding of biology so that patients receive the right drug for their disease at the right dose and the right time. Although the definitions of personalizing vary, they all include the use of different biomarkers driven by a decision-making process in which a diagnostic test is pivotal. Biomarkers include gene expression products, metabolites, polysaccharides, and other molecules such as circulating nucleic acids in plasma and serum, single-nucleotide polymorphism, and gene variants. Ideal biomarkers for use in diagnostics and prognostics, and for drug development and targeting, are highly specific and sensitive [142].

Prostate cancer grows slowly compared to other types of malignancies, which allows it to be an ideal candidate where immunotherapy can be effective. However, various clinical trials by active immunotherapy, passive immunotherapy, adoptive T-cell therapy, and immune checkpoint inhibitors in combination with chemotherapy thus far have only shown modest clinical outcomes in mCRPC when compared with other genitourinary cancers. Different types of mutated proteins exist in a tumor, including KRAS and TP53 [143]. The number of mutations in certain tumors can be used as a tool to predict their response to immunotherapies, anti–PD-1/PD-L1, and anti–CTLA4 monoclonal antibodies [143].

Many mechanisms of immunotherapy resistance have been characterized and more continue to be uncovered. These efforts can improve the quality of medical care for cancer diagnosis and treatment, which improve the quality of life of patients, and finally lead to accurate individualized treatment. Mechanisms of resistance include tumor-intrinsic factors which are changes in anti-tumor immune response pathways, alteration of signaling pathways in tumor cells, and other changes in tumor cells that lead to an inhibitory immunosuppressive microenvironment [144]. And extrinsic factors involve local TME and host-related factors, which can synergize with tumor cells to promote their growth and resistance to ICIs.

There are a variety of immune-suppressive factors in the TME, such as PD-L1, IL-10, hypoxia-inducible factor 1-alpha (HIF-1α), and TGF-β [109]. There is evidence suggesting that an increase in myeloid-derived cells, TAMs, and MDSCs in the TME of prostate cancer is associated with tumor progression [110]. Chronic inflammation induced by the tumor, inflammatory cytokines such as soluble tumor necrosis factor (sTNF), interleukin 1 beta (IL-1β), TGF-β, and IL-10 cause myeloid cells to differentiate into MDSCs, which have been implicated in worsened prognosis and resistance to immunotherapy [111]. To date, there has been only modest demonstrable efficacy of immunotherapy for prostate cancer [112].

Although immune checkpoint inhibitors have shown encouraging clinical responses in certain types of cancer including melanoma, their application to other cancers needs further optimization. Unpredictable efficacy and toxicity of the therapy often become hindrances of successful immunotherapy in many cancers. Various patient responses to the same immunotherapy in patients with different types and stages of cancers have been observed [145]. In addition, patient response depends on multiple factors including intratumor heterogeneity and previous treatment history, which suggests the need for personalized and combination therapy as important future directions for successful immunotherapy.

## 8. Challenges and Future Directions

Nowadays, immunotherapy has risen as part of the arsenal treatment for several cancer types, allowing better outcomes. Prostate cancer, as one of the most common malignant neoplasia, is one of the studied targets for immunotherapy [146]. B7-H3 has been described as a potent suppressor of anti-tumor responses [147]. It is found to be abnormally expressed in benign prostatic tissues as well as prostate cancer. The main difference was thar malignant tissue had higher levels of B7-H3. In 2023, a study reported that B7-H3 was among the most highly expressed immunomodulatory genes in castration-resistant prostate cancer biopsies. Most of the cases (93%) expressed B7-H3, and in patients who developed castration-resistant prostate cancer, B7-H3 expression was frequently expressed at the time of hormone-sensitive prostate cancer diagnosis (97%). Conversion from B7-H3 positive to negative, or vice versa, during progression from sensitive to resistance was uncommon [148].

B7-H3 was upregulated in prostate tumors with DNA repair defect genes, (BRCA2 and ATM), and was associated with high expression of ERG and androgen receptors [148]. Researchers reported that B7-H3 concentrations decreased during neoadjuvant hormone therapy. On the other hand, levels of B7-H3 remained high, contributing to resistance mechanisms in metastatic castrate-resistant cancer to enzalutamide [149].

As a solid tumor, prostate cancer, a significant portion of the mass consists of macrophages, which exhibit M2-like characteristics. These cells play a crucial role in several cancer stages by influencing the tumor’s malignant properties from the initial development to metastasis. A higher presence of macrophages in prostate cancer correlates with more severe clinicopathological features, making them a key focus for new treatments. In advanced metastatic castration-resistant prostate cancer, higher recurrence and mortality rates are associated with an increase in M2-macrophages. The prevalence of M2-macropages increases significantly in elevated Gleason scores compared with healthy tissue. At this point, the evidence underscores the influencing of macrophage plasticity in immunotherapy outcomes [150].

Clinical guides at the international level have a well-defined stock of surgical and clinical managements for prostate cancer in its very diverse stages. Thirty years ago, several of these novel treatments, such as Enzalutamide and Abiraterone, were not even imagined, but the principles for creating them were already known. Today, these products improve the quality of life for many people, something that was not feasible a few decades ago. Even the Da Vinci robotic platform was previously (several years ago) presented as a tool used for the army in helping injured soldiers in order to perform surgeries in a remote way. Nowadays, robotic-assisted surgery is a fact in the clinical arena and increasing every day. The role of immunotherapy is just awaiting the very moment to introduce new management alternatives for prostate cancer.

## 9. Conclusions

Medicine is one of the most dynamic scientific practices. This is because it relies on basic research to propose new treatment strategies and bring them to the clinical setting. Currently, prostate cancer is one of the malignant neoplasms in which the pharmaceutical and innovation industries have invested the most resources. To treat this disease, there is a fairly broad diagnostic and therapeutic arsenal, which allows for diversification of therapeutic options and individualization of cases with increasingly better results.

Beyond its direct anti-tumor effects, immunotherapy holds significant promise for reshaping the clinical management of prostate cancer through improved patient stratification and precision medicine. Advances in tumor immunogenomics, biomarker discovery, and immune profiling are expected to identify subsets of patients who are most likely to benefit from immune-based interventions. This will facilitate rational combination strategies, integrating immunotherapy with androgen deprivation therapy, chemotherapy, RT, or targeted agents to overcome immune resistance and enhance therapeutic efficacy. Such multimodal approaches may not only prolong survival but also improve quality of life by reducing treatment-related toxicity and delaying disease progression.

The role of immunotherapy at this point is ambiguous. When laying our cards on the table, we must accept that health issues do not depend solely on willpower but are an important part of the economy, which implies potential benefits for companies and can often influence the implementation of public health policies. This is the turning point for immunotherapy, as, like other molecules that are now the main therapies on the market, it also went through a period of uncertainty, since good results in experiments are unfortunately not enough to integrate a product into the global therapeutic mix.

Immunotherapy has proven effective in other urological malignancies with good results. As evidence accumulates from clinical trials and real-world data, immunotherapy may shift treatment paradigms toward earlier disease stages, including hormone-sensitive and localized settings. In parallel, sustained investment in basic and clinical research will be critical to address current limitations, such as modest response rates and high costs. In the long term, immunotherapy is poised to become a cornerstone of prostate cancer management, reflecting a convergence of scientific innovation, clinical need, and evolving healthcare priorities.

## Figures and Tables

**Table 1:** Clinical trials of immune checkpoint inhibitors in PCa.

NCT Number	Condition	Interventions	Status
NCT05716295	AST/MST	MDK-703 Checkpoint Inhibitor	Terminated
NCT04954599	Unspecified Adult Solid Tumor	CP-506 Carboplatin Immune checkpoint inhibitor	Recruiting
NCT04717154	CRPC	Ipilimumab Nivolumab	Active, not recruiting
NCT06249750	mCRPC	Immunotherapy Targeted therapy Device: Hyperthermia	Recruiting
NCT02484404	CRC, BreC	Olaparib Cediranib Durvalumab	Active, not recruiting
NCT03673787	AST, GM, mPCa	Ipatasertib Atezolizumab	Active, not recruiting
NCT03792841	mCRPC, PCa	Acapatamab Pembrolizumab Etanercept Cytochrome P450 (CYP) Cocktail	Terminated
NCT03406858	mCRPC	HER2Bi-Armed Activated T Cells Pembrolizumab	Completed, WR
NCT02423928	PCa	Dendritic cell based cryoimmunotherapy Cyclophosphamide ipilimumab	Completed
NCT06022757	HNSCC, UC, PCa, SCLC, NSCLC, CC, OST.	XNW5004 KEYTRUDA^®^ (pembrolizumab) 25 mg/mL Solution for Injection	Recruiting
NCT04631601	mCRPC	Acapatamab Enzalutamide Abiraterone AMG 404	Terminated
NCT03549000	NSCLC, TNBC, PDAC, MSS CRC, OC, RCC, mCRPC	NZV930 PDR001 NIR178	Terminated
NCT04478279	GB, GM, Stage IV Melanoma, BreC, PCa, BraC mBreC, mM, mPCa, Recurrent Melanoma, Recurrent GB, Newly Diagnosed GB	ST101 Temozolomide Radiation	Active, not recruiting
NCT03170960	UC, RCC; NSCLC, CRPC, TNBC, OC, EC, HCC, GC, GEJ, CRC, HNC, DTC, LEC	Cabozantinib Atezolizumab Cabozantinib Cabozantinib	Active, not recruiting
NCT03845166	RCC, HR+ BreC, mCRPC, CRC	XL092 Atezolizumab Avelumab	Active, not recruiting
NCT04404140	CRPC	Ipatasertib Atezolizumab Docetaxel	Terminated
NCT03016312	CRPC	Atezolizumab Enzalutamide	Completed, WR
NCT03821246	PCa	Atezolizumab Tocilizumab Etrumadenant	Recruiting
NCT04751929	PCa, mCRPC	Abemaciclib Atezolizumab	Active, not recruiting, WR
NCT05168618	CRPC, mCRPC	Atezolizumab Cabozantinib S-malate	Recruiting
NCT02814669	CRPC	Atezolizumab Radium-223 Dichloride	Completed
NCT04446117	mCRPC	Cabozantinib Atezolizumab Abiraterone Acetate Enzalutamide Prednisone	Active, not recruiting, WR
NCT05944237	AST	HTL0039732 Capsules HTL0039732 Capsules and atezolizumab infusion	Recruiting
NCT04262154	mPCa	Atezolizumab Abiraterone Prednisone GnRH analog Stereotactic Body Radiotherapy Enzalutamide	Active, not recruiting
NCT04812366	PCa	Apalutamide 60 mg Tab Abiraterone Acetate 250 mg Prednisone 5 mg Tab Docetaxel Niraparib 100 mg Oral Capsule Atezolizumab	Recruiting
NCT02655822	RCC, mCRPC	Ciforadenant Ciforadenant + atezolizumab	Completed
NCT03568656	mCRPC, mBreC, NSCLC, AST	CCS1477 Abiraterone acetate Enzalutamide Darolutamide Olaparib Atezolizumab	Completed
NCT03024216	mPCa	Atezolizumab1200 mg IV Sipuleucel-T	Completed
NCT04471727	SCLC, NEC	Gocatamig Atezolizumab Ifinatamab Deruxtecan (I-DXd)	Recruiting
NCT05253053	AST	TT-00420 Combination Product: Atezolizumab Combination Product: Nab-Paclitaxel	Completed

AST: Advanced Solid Tumor(s); BraC: Brain Cancer; BreC: Breast Cancer; CC: Cervical Cancer; CRC: Colorectal Cancer; CRPC: castration-resistant prostate cancer; DTC: Differentiated Thyroid Cancer; EC: Endometrial Cancer; GB: Glioblastoma; GC: Gastric Cancer; GEJ: Gastroesophageal Junction Adenocarcinoma; GM: Glioblastoma Multiforme; HCC: Hepatocellular Carcinoma; HNC: Head and Neck Cancer; HNSCC: Head and Neck Squamous Cell Carcinoma; HR+ BreC: Hormone Receptor-Positive Breast Cancer; LEC: Lower Esophageal Cancer; mBreC: Metastatic Breast Cancer; mCRPC: metastatic castration-resistant prostate cancer; mM: Metastatic Melanoma; mPCa metastatic prostate cancer; MSS CRC: Microsatellite Stable Colorectal Cancer; MST: Metastatic Solid Tumor(s); NEC: Neuroendocrine Carcinoma; NSCLC: Non-small Cell Lung Cancer; OC: Ovarian Cancer; OST: Other Solid Tumors; PCa: prostate cancer; PDAC: Pancreatic Ductal Adenocarcinoma; RCC: Renal Cell Carcinoma; SCLC: Small-cell; Lung Cancer; TNBC: Triple Negative Breast Cancer; UC: Urothelial Carcinoma; WR: With results.

**Table 2:** Clinical trials of bispecific antibodies in PCa.

NCT Number	Condition	Interventions	Status
NCT05652686	SCLC, LCNEC, NEPC, GEP-NEC, NEC, EP-NEC	Peluntamig (PT217) Carboplatin + Etoposide Paclitaxel Atezolizumab	Recruiting
NCT04104607	CRPC	CC-1, PSMAxCD3	Recruiting
NCT01723475	PCa	BAY2010112	Completed
NCT06095089	AdvPCa	JNJ-78278343 JNJ-87189401	Recruiting
NCT06826768	PCa	REGN5678 Cemiplimab	Recruiting
NCT05646550	RecPCa	CC-1 Infusion	Recruiting
NCT04740034	mCRPC	AMG 340	Terminated, WR
NCT05125016	mCRPC	REGN4336 Cemiplimab REGN5678 Sarilumab	Recruiting
NCT02262910	PCa	ES414	Completed
NCT03927573	NSCLC, PCa, RCC, TCC	GEM3PSCA	Terminated
NCT05032040	OC, ClearCC, EC, CC, mCRPC	Vudalimab	Active, not recruiting
NCT04424641	AST/MST, PCa, EsoC, TNBC, HNSCC, NSCLC, BC, UtC	GEN1044	Terminated, WR
NCT05588609	NSCLC Harboring, *NRG1* Fusion, mCRPC	Afatinib Oral Tablet Enzalutamide Pill Abiraterone acetate tablets MCLA-128	Terminated
NCT05293496	AST, CRPC, Mel, PDAC, HCC, EOC, RCC	Vobramitamab duocarmazine Lorigerlimab	Completed
NCT06999187	TNBC, HER2-BreC, NSCLC, CC, CRPC, PDAC, HNSCC, EC, OC, GC, GEJ, UC	DR-0202	Recruiting
NCT03517488	Mel, BreC, HCC, UC, HNSCC, RCC, CRC, NSCLC, GC/GEJ, EC, Meso, NEC, CC, SCLC, ASCC, CRPC, NPC, Chol, BCC, OC, FTC, Thy, TC, PSCC, VC, OST ^1^, MAN, Non-SCCSG	XmAb20717	Completed
NCT05585034	HNSCC, Mel ^2^, NSCLC Squamous or Non-squamous, UC, Clear RCC, CRPC, EOC, TNBC, CRC	XmAb808 Keytruda (pembrolizumab)	Completed
NCT03849469	Mel, CC, PC, TNBC, HCC, UC, HNSCC, NPC, RCC, NSCLC, SCLC, GC/GEJ, AST/MST, PCa, EOC, FTC, PPC, iChol, ASCC, PSCC, VSCC, CRC, EC	XmAb22841 Pembrolizumab (Keytruda)	Completed
NCT05733351	mCSPC	Abiraterone Docetaxel Enzalutamide Vudalimab	Terminated

AdvPCa: Advanced Prostate Cancer; ASCC: Anal Squamous Cell Carcinoma; AST: Advanced Solid Tumor(s); BC: Bladder Cancer; BCC: Basal Cell Carcinoma; BreC: Breast Cancer; CC: Cervical Cancer; Chol: Cholangiocarcinoma; ClearCC: Clear Cell Carcinoma; CRC: Colorectal Cancer; CRPC: Castration-Resistant Prostate Cancer; EC: Endometrial Cancer; EOC: Epithelial Ovarian Cancer; EP-NEC: Extrapulmonary Neuroendocrine Carcinoma; EsoC: Esophageal Cancer; FTC: Fallopian Tube Cancer; GC: Gastric Cancer; GEJ: Gastroesophageal Junction Adenocarcinoma; GEP-NEC: Gastroenteropancreatic Neuroendocrine Carcinoma; HCC: Hepatocellular Carcinoma; HER2-BreC: HER2-negative Breast Cancer; HNSCC: Head and Neck Squamous Cell Carcinoma; iChol: Intrahepatic Cholangiocarcinoma; LCNEC: Large Cell Neuroendocrine Cancer; MAN: Malignant Adnexal Neoplasms; mCRPC: metastatic castration-resistant prostate cancer; mCSPC: Castration-Sensitive Prostate Carcinoma; Mel: Melanoma; Meso: Mesothelioma; MST: Metastatic Solid Tumor(s); NEC: Neuroendocrine Carcinoma; NEPC: Neuroendocrine Prostate Cancer; NPC: Nasopharyngeal Carcinoma; NSCLC: Non-Small Cell Lung Cancer; OC: Ovarian Cancer; OST: Other Solid Tumors; PC: Pancreatic Cancer; PCa: Prostate Cancer; PDAC: Pancreatic Ductal Adenocarcinoma; PPC: Primary Peritoneal Carcinoma; PSCC: Penile Squamous Cell Carcinoma; RCC: Renal Cell Carcinoma; RecPCa: Recurrent Prostate Cancer; SCCSG: Squamous Cell Salivary Gland Carcinoma; SCLC: Small Cell Lung Cancer; TC: Thymic Carcinoma; TCC: Transitional Cell Carcinoma; Thy: Thymoma; TNBC: Triple-Negative Breast Cancer; UC: Urothelial Carcinoma; UtC: Uterine Cancer; VC: Vulvar Carcinoma; VSCC: Vulvar Squamous Cell Carcinoma; WR: With results. ^1^ With Published Evidence of Anti-tumor Activity with Anti-PD1/PDL1 and/or Anti-CTLA4-directed Therapy. ^2^ Excluding Uveal Melanoma.

**Table 3:** Clinical trials of adoptive cell therapies in PCa.

NCT Number	Condition	Interventions	Status
NCT01140373	PCa	Engineered autologous T cells cyclophosphamide	Active, not recruiting
NCT06236139	CRPC, mCRPC	Anti-STEAP1 CAR T-cells Cyclophosphamide Enzalutamide Fludarabine	Recruiting
NCT05354375	CRPC	CAR-T cell immunotherapy	Recruiting
NCT03873805	CRPC, mCRPC	Autologous Anti-PSCA-CAR-4-1BB/TCRzeta-CD19t-expressing T-lymphocytes Cyclophosphamide Fludarabine Fludarabine Phosphate	Active, not recruiting, WR
NCT05805371	CRPC, mCRPC	Autologous Anti-PSCA-CAR-4-1BB/TCRzeta-CD19t-expressing T-lymphocytes	Recruiting
NCT00105053	PCa	HyperAcute-Prostate Cancer Vaccine	Completed

CRPC: Castration-Resistant Prostate Cancer; mCRPC: metastatic castration-resistant prostate cancer; PCa: prostate cancer. WR: With results.

## Data Availability

No new data were created or analyzed in this study. Data sharing is not applicable to this article.

## Abbreviations

ACT	Adoptive Cell Therapies
ADT	Androgen Deprivation Therapy
APCs	Antigen-Presenting Cells
AS	Active Surveillance
BPH	Benign Prostatic Hyperplasia
bsAbs	Bispecific antibodies
CAFs	Cancer-Associated Fibroblasts
CAR	Chimeric Antigen Receptor
CCR4	C-C Chemokine Receptor 4
CI	Confidence interval
cPAP	Cellular Prostate Acid Phosphatase
CRPC	Castration-Resistant Prostate Cancer
CTLs	Cytotoxic T Cells
Dcr3	Decoy Receptor 3
DCs	Dendritic Cells
DD3	Differential Display Code 3
dMMR	Mismatch Repair deficiency
eTreg	Effector T Regulatory Cells
GM-CSF	Granulocyte Macrophage Colony-Stimulating Factor
GPI	Glycosylphosphatidylinositol
HDRBT	High Dose-Rate Brachytherapy
HGPIN	High-Grade Prostatic Intraepithelial Neoplasia
HIF-1α	Hypoxia-Inducible Factor 1-Alpha
HR	Hazard ratio
ICI(s)	Immune Checkpoint Inhibitor(s)
IHC	Immunohistochemistry
IL-10	Interleukin-10
IL-1β	Interleukin 1 Beta
IMPACT	Immunotherapy for Prostate Adenocarcinoma Treatment
ISH	In Situ Hybridization
ISUP	International Society of Urological Pathology
LncRNA	Long Noncoding RNA
mCRPC	Metastatic Castration-Resistant Prostate Cancer
MDSCs	Myeloid-Derived Suppressor Cells
MHC-I	Major Histocompatibility Complex I
MHC-II	Major Histocompatibility Complex II
MMR	Mismatch Repair
MSI	Microsatellite Instability
MSI-H	High Microsatellite Instability
MUC1	Mucin1
NK	Natural Killer
PAP	Prostate Acid Phosphatase
PCa	Prostate Cancer
PCA3	Prostate Cancer Antigen 3
PFS	Progression-Free Survival
PIN	Prostatic Intraepithelial Neoplasia
PSA	Prostate-Specific Antigen
PSCA	Prostate Stem Cell Antigen
PSMA	Prostate-Specific Membrane Antigen
RT	Radiotherapy
SCLC	Small Cell Lung Cancer
sPAP	Secreted Prostate Acid Phosphatase
STEAP	Six-Transmembrane Epithelial Antigens of the Prostate
sTNF	Soluble Tumor Necrosis Factor
TAAs	Tumor-Associated Antigens
TAMs	Tumor-Associated Macrophages
TCGA	The Cancer Genome Atlas
TCR	T Cell Receptor
TGF-β	Transforming Growth Factor Β
TILs	Tumor-Infiltrating Lymphocytes
TIME	Tumor Immune Microenvironment
TMB	Tumor Mutational Burden
Treg	Regulatory T Cells
